# An Inverse FEM for Structural Health Monitoring of a Containership: Sensor Network Optimization for Accurate Displacement, Strain, and Internal Force Reconstruction

**DOI:** 10.3390/s25010276

**Published:** 2025-01-06

**Authors:** Jacopo Bardiani, Christian Oppezzo, Andrea Manes, Claudio Sbarufatti

**Affiliations:** Department of Mechanical Engineering, Politecnico di Milano, Via G. La Masa 1, 20156 Milano, Italy; christian.oppezzo@mail.polimi.it (C.O.); andrea.manes@polimi.it (A.M.); claudio.sbarufatti@polimi.it (C.S.)

**Keywords:** inverse finite element method, internal force reconstruction, containerships, multi-objective function, structural health monitoring

## Abstract

In naval engineering, particular attention has been given to containerships, as these structures are constantly exposed to potential damage during service hours and since they are essential for large-scale transportation. To assess the structural integrity of these ships and to ensure the safety of the crew and the cargo being transported, it is essential to adopt structural health monitoring (SHM) strategies that enable real-time evaluations of a ship’s status. To achieve this, this paper introduces an advancement in the field of smart sensing and SHM that improves ship monitoring and diagnostic capabilities. This is accomplished by a framework that combines the inverse finite element method (iFEM) with the definition of an optimal Fiber Bragg Gratings-based sensor network for the reconstruction of the full field of displacement; strain; and finally, cross-section internal forces. The optimization of the sensor network was performed by defining a multi-objective function that simultaneously considers the accuracy of the displacement field reconstruction and the associated cost of the sensor network. The framework was successfully applied to a mid-portion of a containership case, demonstrating its effective applicability in real and complex scenarios.

## 1. Introduction

In the context of naval vessels and offshore structures, a crucial aspect has always been safety. Huge economic investments, from millions to even billions of euros in some cases, have been made in military and commercial sector ships; the high costs come from their construction [1] and day-to-day service [2]. This paper focuses on containerships for maritime transportation, a key aspect of international trade, as they represent the most economical and sustainable way to move large volumes of essential goods and finished products over long distances.

The literature offers many studies, ranging from risk analyses [3] to statistical ones [4], highlighting how cargo ships are involved in the highest percentage of incidents due to collisions, groundings, or hull failures [4]. Another aspect to consider is the harsh environmental conditions [3], which can cause damage and defects through various phenomena such as fatigue, corrosion, or finally overloading [5].

To ensure the safety of such complex structures, there are two main approaches: maintenance through non-destructive testing (NDT) or structural health monitoring (SHM). Traditional NDT methods differ in terms of the physical principle used and their field of application, such as simple visual inspection, ultrasound, or radiography [6]. In general, they are precise and reliable techniques for detecting damage, but they share the same flaws. Specifically, all NDT methods are implemented according to a maintenance schedule, not necessarily when needed. Furthermore, they can become very expensive over the structure’s lifetime.

In this scenario, the application of SHM is proposed as a more viable alternative, capable of retrieving data in real time or on demand to provide a diagnosis and, in some cases, even a prognosis [7]. What makes SHM a better choice compared to NDT is that the maintenance schedule is not fixed but based on real-time recorded data [8], leading to lower service costs while at the same time ensuring greater safety. As reported in [7], structural monitoring techniques vary depending on the applied physical principle (vibration-based or low-frequency electromagnetics) or the sensors implemented (fiber optics, electrical resistance, or piezoelectric sensors).

In the maritime field, researchers have considered so-called hull structural health monitoring (HSHM) as an important case study to investigate [5]. Several real-world applications have already been implemented, such as the structural health monitoring of naval ship hulls [9,10], the monitoring of a Panamax containership in [5], and monitoring for the Royal Norwegian Navy [8]. In the literature, there has been growing interest in HSHM as a precaution against ships’ structural failures, despite challenges related to hostile environmental conditions, and the complexity and size of structures [11].

A state-of-the-art review of HSHM [12] provided an overview of the gaps and inefficiencies in many current SHM systems. It highlighted the inability to account for overly complex structures and boundary conditions, while also emphasizing the need for accurate information on applied loads, many of which are caused by winds and waves, so with stochasticity and uncertainty in their estimation [13,14]. Moreover, some of these HSHM systems are not suitable for real-time use due to the time-consuming analysis required.

To overcome these challenges, Kefal et al. [15] proposed for the first time in the literature the application of the inverse finite element method (iFEM) for monitoring a square stiffened plate that represents a portion of the side of a typical tanker with longitudinal and transverse framing, using Fiber Bragg Gratings (FBG). This case was particularly relevant as naval structures primarily consist of stiffened plates. Within this context, the iFEM algorithm emerged as a valid choice to perform HSHM. Introduced by Tessler and Spangler [16,17], it is a model-based approach capable of reconstructing the entire displacement field (also called shape sensing) based on the minimization of a weighted error functional, defined as the difference between the measured strains and those numerically formulated. The benefits of iFEM include high computational efficiency, allowing for real-time applications [18]; the lack of a need to know the material and the applied load on the structure [18]; and its simple implementability, even for complex structures [19,20,21].

The iFEM approach requires experimental strain data to be implemented, and the quality and quantity of the measured values remain crucial aspects for obtaining appropriate reconstruction of the displacement field of the structure of interest [10]. To make structural monitoring economically feasible through the iFEM technique, the introduction of a sensor network capable of providing sufficiently accurate results is extremely necessary.

The factors to consider in relation to the sensor network to be put within the structure are the techniques for extrapolating missing strain data and the sensor technology used. Among the extrapolation algorithms described in the literature, there are those based on physical principles [22] and data-driven approaches, such as the smoothing element analysis (SEA) [20]. Additionally, there are simpler solutions based on polynomial functions, like the one used in this study. Regarding the sensor technology to be implemented, many technologies have been proposed in the maritime field [23], but fiber optic sensors stand out as one of the most versatile and advantageous for the structural monitoring of ship hulls, mostly due to their ability to withstand harsh environments, immunity to electromagnetic interference, and reduced cabling installation costs when employing wavelength multiplexing and multi-fiber cables [8].

The robustness of the iFEM algorithm allows for achieving results with an adequate level of precision, even when using a limited number of sensors on complex structures. This has already been confirmed by other studies, such as those involving a tapered (wing-shaped) sandwich laminate [20] and cylindrical marine structures [21].

In this paper, a comprehensive framework for implementing HSHM on the midsection of a containership is proposed, leveraging the iFEM approach. This framework addresses a critical gap in the literature, where a rigorous, optimized, sensor-based monitoring strategy for complex naval structures, such as containerships, is lacking. Unlike current applications of iFEM, which are often limited in scope or complexity, the proposed approach is tailored to accurately reconstruct the displacement field, strain field, and cross-section internal forces in realistic and intricate structures like containerships. Within this framework, the problem of the design of a high-performance sensor network with a minimal number of sensors, strategically positioned to capture essential data with high accuracy, is addressed. The sensor layout is initially informed by a gradient trend analysis and then optimized via a multi-objective function that balances cost efficiency with monitoring quality.

The manuscript is organized as follows. A review of the iFEM methodology is reported in Section 2, while Section 3 globally describes the methodology used in this framework. Section 4 focuses on the application of the entire methodology to a real case study, represented by a portion of a containership. All the results are provided in Section 5, and the conclusions of the work and possible future developments are stated in Section 6.

## 2. Inverse Finite Element Method Review and Internal Force Reconstruction Formulation

A summary of the iFEM procedure for displacement and strain field reconstruction is provided in this section, while a detailed formulation is available in [5,24,25] for the interested reader. The iFEM approach requires the discretization of a model into inverse finite elements to be implemented, such as in the traditional FEM approach. In the literature, three alternatives have been presented: a three-node flat inverse shell element [26] (known as iMIN3); an inverse quadrilateral smoothing four-node flat shell element (iQS4) [24]; and an eight-node curved inverse-shell element, referred to as iCS8 [21]. In the comparative study of these inverse elements presented in [27], it is shown that the iQS4 element is the most suitable in cases where the structure is composed of plates, as in the case study investigated here. This led to the choice of iQS4 elements for the discretization of the ship model.

Once the structural model is created, the standard iFEM approach [16,17] involves calculating the displacement field from input strain measurements by minimizing the least-square functional of Equation (1), which is defined as the error between the input strain field measured by sensors $\left(\cdot^{\varepsilon }\right)$ and its numerical formulation $\left(\cdot \left(u\right)\right)$, which is a function of the unknown nodal displacements u. Both the input and numerical strain fields are separated into three main components: the membrane e, the bending k, and the transverse shear g strain contributions. Thus, the formulation of the *i*-th inverse element can be defined as follows:

(1) $$\Phi_{i}\left(u^{i}\right)={\left\|e\left(u^{i}\right)-e_{i}^{\varepsilon }\right\|}_{W_{m}^{i}}^{2}+{\left\|k\left(u^{i}\right)-k_{i}^{\varepsilon }\right\|}_{W_{b}^{i}}^{2}+{\left\|g\left(u^{i}\right)-g_{i}^{\varepsilon }\right\|}_{W_{s}^{i}}^{2}$$

where ${\left\|\cdot \right\|}_{W}^{2}$ is the squared weighted Euclidean norm with the weight matrix W. Specifically, $W_{m}^{i}$, $W_{b}^{i}$, and $W_{s}^{i}$ are diagonal matrices of weights for the membrane, bending, and transverse shear strain contributions, respectively.

However, the previous formulation has an inherent limitation in managing the weight matrices W. When defining the matrices $W_{m}^{i}$, $W_{b}^{i}$, and $W_{s}^{i}$ for the various strain components of the *i*-th inverse element, the respective values are linked to both sides of the element, without the possibility of differentiation between the top and bottom sides. This constraint generally results in a greater error in the displacement field reconstruction [28] because these weight matrices ensure consistency between numerical and experimental strain measurements, especially in the case of sparse sensor networks. Consequently, when only one side of the element is measured, it would be ideal to assign a different weight to the actual measured data and the missing one.

**Figure 1:**
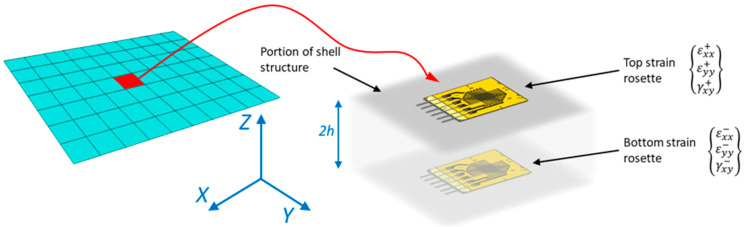
Discrete sensor placement on both the top and bottom surfaces of the shell structure (adapted from [32]).

As an alternative, this paper proposes the application of a new formulation [28], based on a different reprocessing of the same variables, to decouple the behavior between the top and bottom sides of the inverse element. The in-plane contributions, e and k, can be reformulated into a single in-plane strain parameter according to Equation (2). Here, z represents the coordinate along the thickness of the element, while $z^{+}$ and $z^{-}$ refer to the surfaces of the element with z coordinates equal to h and −h, respectively, with reference to the local coordinate system shown in Figure 1.

(2) $$\varepsilon_{p}\left(z,u\right)=\left[\begin{array}{ccc} 1 & 0 & 0\\ 0 & 1 & 0\\ 0 & 0 & 1 \end{array}\;\begin{array}{ccc} z & 0 & 0\\ 0 & z & 0\\ 0 & 0 & z \end{array}\right]\left[\begin{array}{c} e(u)\\ k(u) \end{array}\right]$$

Thus, the functional according to the new formulation of the *i*-th inverse element can be defined as follows:

(3) $$\Phi_{i}\left(u^{i}\right)={\left\|\varepsilon_{p,i}\left(z^{+},u^{i}\right)-\varepsilon_{p,i}^{+}\right\|}_{W_{+}^{i}}^{2}+{\left\|\varepsilon_{p,i}\left(z^{-},u^{i}\right)-\varepsilon_{p,i}^{-}\right\|}_{W_{-}^{i}}^{2}\;\;+{\left\|g_{i}\left(u^{i}\right)-g_{i}^{\varepsilon }\right\|}_{W_{s}^{i}}^{2}$$

where $\left(\cdot^{+}\right)$ and $\left(\cdot^{-}\right)$ are, respectively, the input strain fields measured by sensors on the top and bottom sides of the element, with reference to the local coordinate system (Figure 1). It can be observed that for both formulations (1) and (3), the underlying principle is the same: minimization of the respective least-squares functional, defined as the error between the input strain field measured by sensors and its numerical formulation. The only differences are the reformulation of the in-plane strain variables and the definition of the new weight matrices $W_{+}^{i}$ and $W_{-}^{i}$, while the descriptions related to the component g and $W_{s}^{i}$ remain unchanged. The main advantage of the new formulation (3) is the increased flexibility in assigning weights to the matrices W, based on the deformation component; the element; and now also, the face of the element.

The matrices $W_{+}^{i}$, $W_{-}^{i}$, and $W_{s}^{i}$ are associated with each side of the *i*-th inverse element, the diagonal matrices of weights for the in-plane top, in-plane bottom, and transverse shear strain contributions, respectively. Note that each matrix W contains different weights along the main diagonal, corresponding to the strain components along the *x*-axis, the *y*-axis, and the in-plane shear, apart from $W_{s}^{i}$, which includes only the first two components, relative to the element’s local reference system (Figure 1). The corresponding diagonal values are assigned based on the specific case to which they belong, as shown in Table 1. It should be noted that these values are specific to the case study investigated in this work and may differ for other cases.

The weights in Table 1 vary depending on the quality of the strain data considered. Starting from a reference value of one when the measurement is available, down to very low values in the case of missing data (e.g., $10^{-4}$). The only situation not accounted for in Table 1 occurs when the strain component is measured from only one side of the element. In this case, it is proposed in this paper to use the same measurement from the opposite side, effectively approximating the strain component as constant through the thickness. The accuracy of this approximation is appropriately weighted by the matrix W, whose values cannot be determined a priori but must be evaluated based on the specific case considered (further details on this scenario are provided in Section 4.2).

In the broadest scenario, the input strain formulation is derived from strain measurements taken on the structure. Sensors are typically placed on the external surfaces of the component for easier installation and maintenance, although applications with embedded sensors are also feasible.

For example, consider a couple of strain gauge rosettes applied on the two external sides of the shell, as shown in Figure 1. The in-plane top and in-plane bottom strain components associated with the *j*-th sensors’ location within the *i*-th inverse element can be defined as follows:

(4) $$\varepsilon_{p,ij}^{+}={\left\{\begin{array}{c} \varepsilon_{xx}^{+}\\ \varepsilon_{yy}^{+}\\ \gamma_{xy}^{+} \end{array}\right\}}_{j}\;\varepsilon_{p,ij}^{-}={\left\{\begin{array}{c} \varepsilon_{xx}^{-}\\ \varepsilon_{yy}^{-}\\ \gamma_{xy}^{-} \end{array}\right\}}_{j}$$

The strain component ***g***, on the other hand, cannot be directly measured or computed from the surface strain components. However, since its contribution can be neglected in most of engineering applications, particularly in structures modelled by thin shells, such as the containership analyzed, as well as in other offshore structure studies [5,15,29], the ***g*** formulation is neglected hereon.

For precise reconstruction of the displacement field, it is necessary to have the input strain field available for all elements of the structure. However, this is generally impractical in real-world applications. To address this limitation, strains can be extrapolated in areas where physical sensors are unavailable. This can be achieved through methods such as polynomial fitting, like the one used in this study, or SEA [20,30,31], utilizing either data-driven or physics-based approaches. Data-driven methods extrapolate strains solely based on the acquired strain field, resulting in a more continuous and smooth output across the entire domain. Conversely, physics-based strain extrapolation combines the acquired strain data with knowledge of geometrical discontinuities and their analytical formulations to provide a more accurate strain field, especially in the presence of discontinuities.

The numerical strain component formulation is based on the element’s shape functions; thus, it is computed through Equation (5), where $B^{m}$, $B^{b}$, and $B^{s}$ are matrices containing the derivative of the shape functions [5,24].

(5) $$e\left(u^{i}\right)=B^{m}u^{i}\;k\left(u^{i}\right)=B^{b}u^{i}\;g\left(u^{i}\right)=B^{s}u^{i}$$

After some mathematical passages applied to Equations (4) and (5) inside Equation (3), by employing a conventional assembly procedure that accounts for the contribution of each inverse element and aims to minimize the overall least-squared functional, the problem can be formulated as follows:

(6) KU=F

where K is a matrix resembling the stiffness linking the global displacement field U with the vector F containing the input strain field contribution.

Nonetheless, the matrix K is singular, and if left unconstrained, it will result in rigid motion of the structure. Therefore, after establishing appropriate boundary conditions, the free (unconstrained) nodal displacement can be determined. Subsequently, once the displacement field has been calculated, the numerical strain field $\varepsilon_{iFEM}$ can be computed using Equation (5), similarly to any direct FEM approach.

What has been explained so far is that an inverse model $M_{iFEM}$ is available for real-time numerical prediction of the strain field $\varepsilon_{iFEM}$ as a function of the vector of strain measurements $\varepsilon_{in}$ without requiring any a priori knowledge of loads or material properties since only strain–displacement relationships are involved in the calculations.

**Figure 2:**
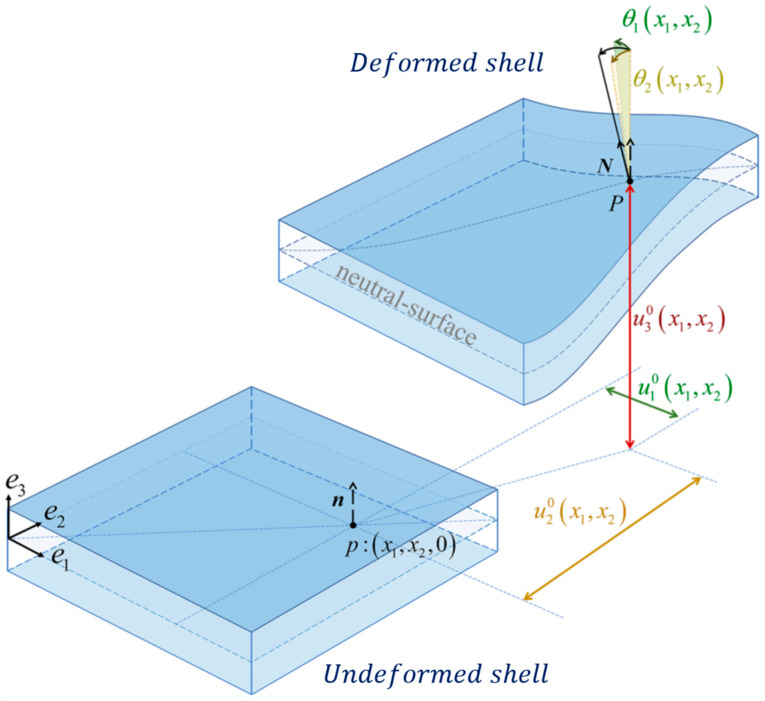
The kinematics of a shell structure. Adapted from [33].

The reconstruction of the displacement field using the iFEM algorithm provides valuable information for monitoring ship structures, but it is not the only data that can be retrieved. The second part of this section offers an overview of the strategy employed in this study to reconstruct the internal forces within transverse structural sections based on iFEM results. This approach leverages the available material properties and the reconstructed strain field. To calculate these sectional internal forces, the authors refer to the traditional plate/shell theories (Kirchhoff–Love theory or the Reissner–Mindlin theory), as shown in Figure 2.

For this case, the classical vector of sectional strains $\varepsilon ={\left[\varepsilon_{1},\varepsilon_{2},\gamma_{12},\kappa_{1},\kappa_{2},\kappa_{12},\gamma_{1},\gamma_{2}\right]}^{T}$ can be defined, where $\varepsilon_{1},\varepsilon_{2},\gamma_{12}$ are the membrane strains; $\kappa_{1},\kappa_{2}$ are the bending curvatures; $\kappa_{12}$ is the twisting curvature; and finally, $\gamma_{1},\gamma_{2}$ are the transverse shear strains.

Accordingly, the vector of the generalized stresses ${\left[N_{1},N_{2},N_{12},M_{1},M_{2},M_{12},Q_{1},Q_{2}\right]}^{T}$ can be defined as follows:

(7) $$\left(N_{1},N_{2},N_{12},Q_{1},Q_{2}\right)=\int \left(\sigma_{1},\sigma_{2},\tau_{12},\tau_{13},\tau_{23}\right){dx}_{3}$$

(8) $$\left(M_{1},M_{2},M_{12}\right)=\int \left(\sigma_{1},\sigma_{2},\tau_{12}\right)x_{3}{dx}_{3}$$

Sectional stiffnesses/flexibilities are defined to relate sectional generalized stresses to sectional strains. For a uniform thick shell with an isotropic material, such a relation can be expressed as follows:

(9) ε1ε2γ12κ1κ2κ12γ1γ2=1Eh·1−ν0−ν100021+ν00012Eh3·1−ν0−ν100021+ν0001kGh·1001N1N2N12M1M2M12Q1Q2

where k is the well-known shear factor; E, the Young’s modulus; G, the shear modulus; and finally, h, the thickness of the plate. In contrast, for a shell with complex material and structural compositions, its equivalent stiffness/flexibility matrix can be fully populated, which is usually difficult to solve directly using a theoretical analysis.

The previous procedure is used to reconstruct the sectional generalized stresses of the model under investigations. Within this passage, we assumed linear elastic behavior of the structure. For a given section of interest, the corresponding internal forces are calculated as the sum of contributions from each element intersected by the section. Starting from the strain field $\varepsilon_{iFEM}$ reconstructed by the iFEM, the contributions ${\left[N_{1},N_{2},N_{12},M_{1},M_{2},M_{12},Q_{1},Q_{2}\right]}^{T}$ of individual elements are determined by applying Equation (9). Finally, the internal forces of the section considered are obtained as the sum of the products of the generalized stresses and the respective lengths of each element.

## 3. Research Framework Methodology

**Figure 3:**
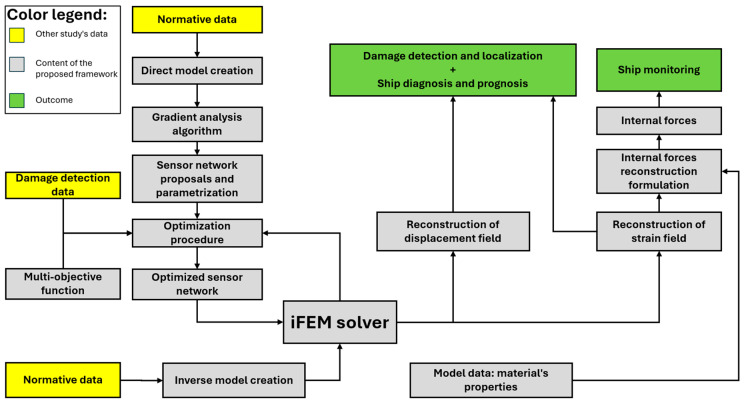
Workflow chart of the framework implemented in the presented research.

The proposed framework for implementing SHM on the containership is outlined in Figure 3 and consists of the following steps:

- Direct FE model creation: a high-fidelity finite element (FE) model, representing the mid-portion of the containership, is created. Data related to the ship’s geometry, boundary and loading conditions are obtained from ship regulations and similar studies [34,35,36]. The direct model is assumed to be subjected to a single load corresponding to the vertical longitudinal bending moment, for simplicity. This model provides all necessary numerical strain measurements as input for the iFEM, due to the unavailability of experimental strain. Additionally, it is used as a reference for evaluating the error in the different reconstruction results generated by the iFEM and is also used as input for the gradient algorithm, offering insights for the preliminary design of the sensor network.
- Inverse FE model creation: an inverse model that shares the same geometry, boundary conditions, and mesh size as the direct model, but without information on material properties or loading conditions, is created. The inverse model is used within the iFEM approach for full-field displacement and strain reconstruction, utilizing data from a proper sensor network as input.
- Gradient algorithm: establishing a sensor network is the key factor that makes the SHM application on the containership economically feasible, but it is also the most challenging aspect to define. The goal of the sensor network is to maintain accuracy in the results while minimizing the number of sensors required. To determine the most suitable sensor locations, a dedicated algorithm was introduced to analyze the strain gradient across the containership, based on data derived from the direct FE model. The gradient algorithm provides the locations of the centroids for the minimum number of elements in the inverse model that require the presence of a sensor to ensure an accurate reconstruction of the strain field.
- Sensor network proposals: the locations where sensors should be put within the structure (identified in the previous step) are determined without accounting for practical constraints associated with the chosen sensor type, specifically FBGs in this case. Therefore, these positions are not directly applicable for constructing a sensor network; however, they serve as guidelines, based on which a realistic, implementable solution has been proposed. In this study, the proposed solution consists of a serpentine pattern of FBGs, parameterized based on the distance between the curves and subsequently optimized according to this parameter within the dedicated procedure.
- Optimization procedure: the previous parametrized sensor network configurations are compared and evaluated using the results from the iFEM approach, with the goal of identifying the optimal one. The optimization procedure is performed graphically using the multi-objective function (see Equation (15)), which integrates both the costs associated with the sensor network and the accuracy of the displacement field reconstructed by the iFEM.
- Internal forces reconstruction: starting from the strain field $\varepsilon_{iFEM}$ reconstructed by the iFEM approach and considering the implementation of the optimized sensor network on the inverse model, the internal forces in various sections of the ship are obtained, as described in Section 2, from the weighted sum of the contributions ${\left[N_{1},N_{2},N_{12},M_{1},M_{2},M_{12},Q_{1},Q_{2}\right]}^{T}$ derived through Equation (9).

## 4. Case Study

This section provides a detailed discussion of the proposed framework as applied to a containership case study. Beginning with a description of the FE model used to represent the mid-section of the ship, it outlines the procedure for developing and optimizing the FBG-based sensor network. This is followed by the application of the iFEM approach to reconstruct the displacement and strain fields, and sectional internal forces.

### 4.1. Direct FE Model

**Figure 4:**
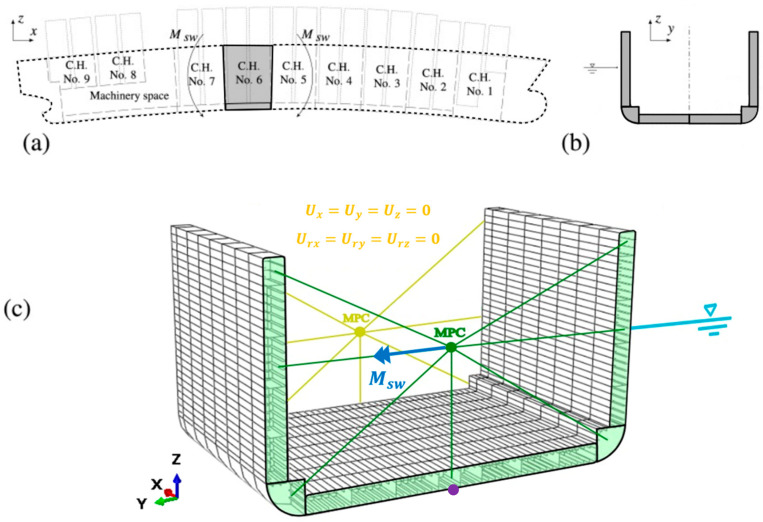
Three-dimensional schematic representation of the benchmark case study under investigation (adapted from [37]): (**a**) longitudinal scheme, (**b**) cross-section, (**c**) structural model.

This paragraph deals with the creation of a high-fidelity numerical FE model (called direct), necessary for implementing the proposed framework. The geometry considered corresponds to part of the double bottom of the midship region of a containership with length $L_{B.P.}=353.0\;\left[m\right]$, breadth $B=51.0\;\left[m\right]$, depth $D=29.9\;\left[m\right]$, and designed draught $T=14.5\;\left[m\right]$. Figure 4 contains a schematic representation of the employed case study including the geometry as well as the applied load. In the interest of brevity, the reader is referred to [37] for detailed structural information which is not contained here; all FE calculations were made in ABAQUS CAE version 6.23 [38].

**Figure 5:**
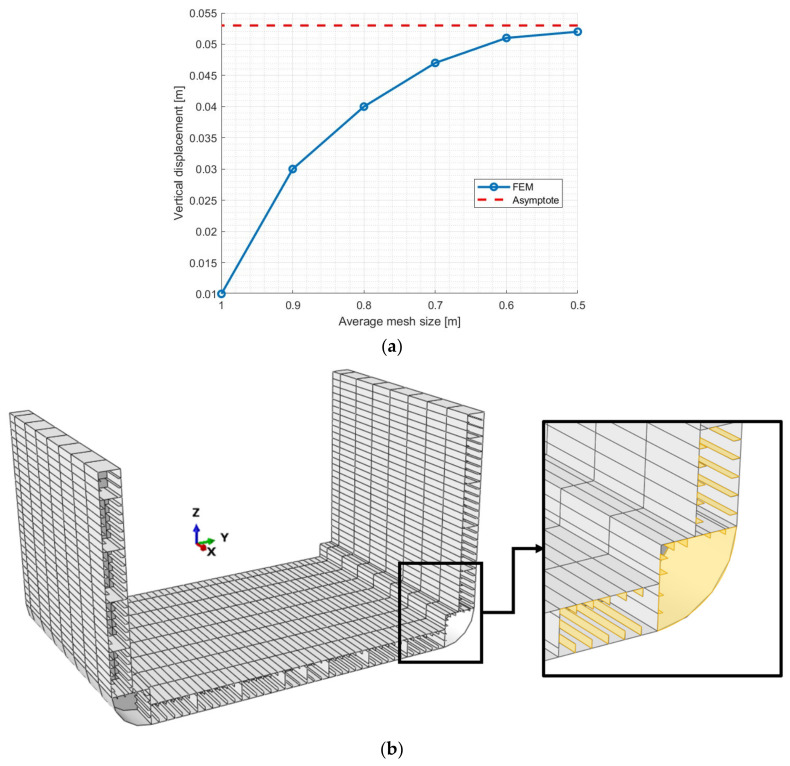
(**a**) Example of mesh convergence of the direct model for the vertical displacement profile of the violet point in Figure 4c. (**b**) Indication of the surfaces not subjected to the strain extrapolation procedure (highlighted in yellow).

The model is composed of 45,060 4-node quadrilateral shell elements with full integration [15,29]. The average mesh size of the numerical model is 500 mm, while no refinement strategy has been adopted. The mesh size was determined after performing a mesh convergence process to ensure that the displacements in the most important locations are accurately captured. Just as an example, Figure 5a shows the direct FEM convergence chart for the vertical displacement profile of the violet point in Figure 4c. Aside from that, the computational cost associated with the analyses was considered.

The remaining aspects to be defined include the material, boundary, and loading conditions. Regarding the material, typical naval mild steel properties are used, namely $E=209\;\left[GPa\right]$, Poisson’s ratio $\upsilon =0.3\left[-\right]$, and density $\rho =7850\;\left[kg/m^{3}\right]$ [39,40].

The choice of boundary and loading conditions is described as follows. The BCs were inspired by those considered in [34,36], which specify that the planes at the ends (fore and aft) of the FEM portion model must remain flat under the action of the bending moment, while these cross-sections must be able to rotate freely (in the case of longitudinal resistance only). For this purpose, all nodes associated with the continuous longitudinal stiffeners at the two ends of the model must be rigidly connected to an independent point. The independent point was placed along the centerline at a height close to the position of the neutral axis (at the coordinate $Z=8535\;\left[mm\right]$). These independent points are connected to the model by rigid links through the usage of a multipoint constraint (MPC, type “beam” in ABAQUS CAE) and can only rotate freely along the *y*-axis, where the longitudinal vertical bending moment $M_{sw}$, the unique bending action considered for simplicity, is applied.

Due to the internal requirements of the iFEM code used in the present framework, instead of applying two bending moments in the MPC points at the ends of the model, allowing the ends to rotate freely around the *y*-axis, it was decided to apply a fixed boundary condition at one section and leave the degrees of freedom free at the other, achieving the same bending behavior results, as schematically shown in Figure 4c. In this case, the required vertical bending action $M_{sw}$ was applied only to the independent point of the green end section shown in Figure 4c.

As already mentioned, for simplicity, only the longitudinal vertical bending moment on the hull has been considered in this study as it is the most impactful on the global effects of the ship girder; the calculation was possible through the implementation of empirical formulations proposed by the International Association of Classification Societies (IACS) [35,36]. The equation used here for the sagging load condition is the following:

(10) $$M_{sw}=-220{\cdot F}_{M}\cdot f\cdot C_{w}{\cdot L}^{2}\cdot B\cdot \left(C_{B}+0.7\right)\cdot 10^{-3}=9\cdot 10^{9}\left[kNm\right]$$

where $F_{M}$ is the factor of distribution of the bending moment over the length of the ship and can be obtained from tables, f is the navigation constant (from tables), $C_{w}$ is the wave parameter, L is the sizing length of the vessel in meter, B is the maximum width out of the frame amidships, and $C_{B}$ is the total fineness coefficient of the vessel, calculated out of planking, therefore including the factor $k_{C}=1.006$. No information about the precise values is reported here for simplicity, since IACS formulations are well known in the field of marine engineering design. It is important to note that the previous formula proposed by the IACS accounts not only for the bending moment in still water but also for the contribution due to waves. The application of the $M_{sw}$ action on the independent node of the MPC, as described above, allows for the application of the bending action, distributing it across the entire cross-section highlighted in green in Figure 4c. The evaluation of additional loading conditions is left to future studies.

The described FE numerical model corresponds to the direct FE model, on which a finite element analysis (FEA) is performed to obtain the strain and displacement fields that the containership undergoes, in the absence of experimental data. This strain field is then used as input for strain measurements in both the application of iFEM and the implementation of the gradient algorithm. In parallel, the displacement field generated by the direct FE model serves as a reference to assess the accuracy of the displacement field reconstruction generated by iFEM.

### 4.2. Inverse FE Model

The iFEM approach, described in detail in Section 2, requires the creation of an inverse FE model, where the sensor network can be defined and the weights associated to the matrices $W_{m}^{i}$, $W_{b}^{i}$, and $W_{s}^{i}$ assigned. The inverse model shares the same geometry, boundary conditions, and average mesh size as the direct model (for simplicity), with the difference that its mesh is composed of iQS4 elements. Additionally, this model does not include material or loading information for the containership, as these are unnecessary information for iFEM. Regarding the assignment of weight coefficients, this process has been carried out for each side of every element of the inverse model, according to Table 1 and the procedure described in Section 2.

Regardless of the proposed sensor network (investigated subsequently), certain surfaces of the inverse model have been excluded as sensor-applicable surfaces due to practical limitations associated with the chosen sensor type, as well as the procedure for extrapolating missing strain data.

The surfaces shown in Figure 5b represent all surfaces not subjected to the strain extrapolation procedure due to their low structural relevance for monitoring [41] and the inaccuracy of data obtained using the extrapolation method applied in this study, which is based on polynomial functions. It is important to underline that in Figure 5b, not only are the surfaces highlighted in the zoomed-in area excluded from the strain extrapolation, but this consideration also applies to all elements of the same surface type throughout the structure.

For the inverse mesh elements on the remaining surfaces, except for those where a sensor is assumed to be present, the extrapolation process has been applied. This process is initially performed on surfaces partially equipped with sensors, with the aim of extending the collected data, through a subsequent spatial extrapolation process, to the unmonitored surfaces as well.

The only scenario not covered in Table 1 is the assignment of weight coefficients in the locations where a sensor is positioned on only one side of an element. This paper proposes associating the same measured strain component to the opposite element’s side, assuming a constant distribution through the thickness, while assigning a unit value to the weight coefficients on the sensor side and a value $\lambda_{side}$, yet to be defined, to the opposite side (where the sensor is missing). Differentiating the weight coefficients inside W by implementing Equation (3) allows for greater flexibility and consistency in assigning weights. In general, assuming the same measurement on both sides of the element does not necessarily lead to an accurate approximation; however, for the purposes of the iFEM approach, the essential requirement is to assign values to the W matrices that represent the quality of the data. Nevertheless, for the case under examination, this assumption is appropriate since the entire inverse FE model was created as a thin-plate structure, with thicknesses several orders of magnitude smaller than the overall dimensions of the structure.

**Figure 6:**
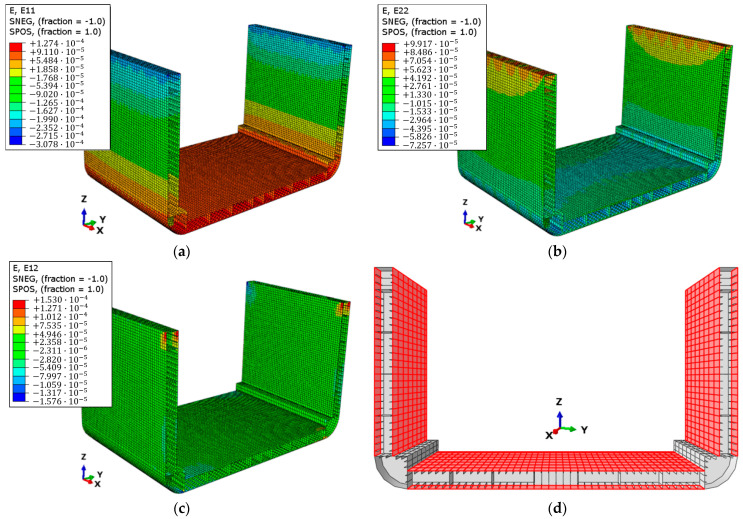
Direct model FEA results: (**a**) E11, normal strain field along x-direction, (**b**) E22, normal strain field along y-direction, (**c**) E12, shear strain field on x, y-plane. (**d**) Indication of sensor-applicable surfaces.

In this study, the value of $\lambda_{side}$ has been estimated according to the strain field in Figure 6a–c, provided by the direct FE model. The differences in strain components between the top and bottom sides of the various surfaces were found to be less than 1.5%. This difference aligns with the definition of the direct model, as the only load applied is a longitudinal vertical bending moment (Figure 4). Consequently, surfaces with a normal parallel to the moment vector direction show negligible differences between the two sides of the elements. Conversely, on orthogonal surfaces, while the variation is slightly higher, it remains very small as a percentage due to the minimal distance difference from the neutral axis between the two sides, which does not exceed 0.24%. Through an iterative procedure based on iFEM simulations, a $\lambda_{side}$ value of 0.9 was determined to be optimal, as it ensures convergence toward a minimal error. The error was evaluated as the percentage difference in the displacement field magnitude between the results of the direct model FEA and the iFEM reconstruction.

### 4.3. Sensor Network Proposal, Parametrization, and Optimization

Implementing SHM systems on large and complex structures like containerships generally requires numerous sensors to accurately assess its health status, especially in the absence of an effective sensor placement strategy. However, installing sensors across every area of a containership is not cost-effective. Therefore, developing an optimized sensor network becomes the best compromise. Although various algorithms and sensor deployment techniques are available in the literature, the current state of research shows limitations due to the fragmented and application-specific approaches often employed by researchers [42].

The proposed framework incorporates a fiber optic sensor network, developed and optimized for the case studied but generally adaptable to any real-world scenario. For the iFEM approach, strain field measurements are required as input. Among the available sensor types [6], fiber optics were chosen for their ease of installation, compact size, and ability to operate in harsh environments. Additionally, they can be strategically attached to the ship’s structure in a tailored pattern, enabling extensive coverage of critical areas with an interconnected network of fibers [8,15]. The specific type of fiber optic supposed in the framework is Draw Tower Gratings (DTG). The definition of an effective sensor network in this paper was established through the following passages: initially, an algorithm based on the strain gradients developed on the structure identifies the positions where sensors need to be installed (areas with higher gradients require a greater sensor density compared to those characterized by lower values, to ensure an accurate reconstruction of the displacement field); then, based on these positions, a serpentine fiber optic pattern (FOP) is proposed as a sensor network, which is parametrized based on the distance between the curves. After that, the parametrized sensor networks are evaluated through a multi-objective function (see Equation (15)) to identify the optimal configuration.

#### 4.3.1. Gradient Algorithm Analysis

Creating a high-performance sensor network requires the use of specific techniques or algorithms, especially for complex and intricate cases like a containership. Among the existing families of algorithms, genetic algorithms are a valid choice for ship sensorization due to their versatility and general applicability. Their functioning principle involves the iterative and random generation of new solutions, based on those from the previous generation, until an effective population of solutions is obtained [43,44]. However, genetic algorithms tend to be slow and computationally expensive. As an alternative, this paper proposes a simple deterministic algorithm, based on the gradient analysis of strains (gradient algorithm). The principle of the gradient algorithm is to identify areas of the structure characterized by a minimal and/or constant strain gradient, to assess the possibility of not placing sensors in such zones. This is because areas with low or constant strain gradients, respectively, show minimal variation and a more linear trend in the values, making them easier to interpolate accurately. As a result, the gradient algorithm provides the locations of the centroids for the minimum number of elements in the inverse model that require the presence of a sensor to ensure accurate extrapolation and reconstruction of the strain field. These locations are ultimately used as a reference for proposing a sensor network pattern to be parametrized (further details in Section 5).

In the proposed framework, the gradient algorithm was used due to its low computational cost and the versatility it offers in evaluating the gradient of any field of values across the entire structure under analysis. The gradient algorithm is based on the following assumptions: availability of information regarding the geometry, material properties, and boundary conditions of the structure in question; knowledge of the types of loads applied; and their respective magnitudes. These data are necessary to obtain a representation of the strain field of the structure to be analyzed. For this case, the direct model and the corresponding FEA results (Figure 6a–c) were used as reference data.

**Figure 7:**
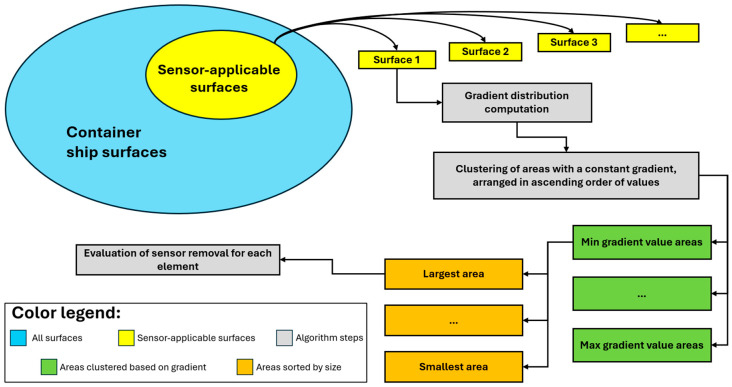
Workflow chart of the framework to sensorize the containership.

The gradient algorithm is outlined in Figure 7 and consists of the following steps:

I. Identify the sensor-applicable surfaces: Among all surfaces of the structure, the ones deemed suitable for sensor placement are the inner faces of the three bulkheads highlighted in Figure 6d. These surfaces, being the largest, allow for an interconnected fiber network to be applied, offering extensive coverage of the ship’s critical areas. The remaining surfaces were excluded for logistical and operational reasons, including exposure to seawater, the presence of cargo constraints, and the practicality of installing an interconnected fiber network on a single plane, thereby avoiding installation on transverse structural elements.
II. Gradient distribution computation: Based on the results from the direct model FEA, the equivalent strain value $\varepsilon_{eq}$ is calculated for each element on the surfaces selected from step I. This is carried out using Equation (11) based on all the strain components visible in Figure 6a–c. The distribution $\varepsilon_{eq}$, obtained on each of the sensor-applicable surfaces, represents all measurable strain components on each FE.
  (11) $$\varepsilon_{eq}=\frac{1}{\sqrt{2}}\cdot \sqrt{{\left(\varepsilon_{xx}-\varepsilon_{yy}\right)}^{2}+{\left(\varepsilon_{xx}\right)}^{2}+{\left(\varepsilon_{yy}\right)}^{2}+6{\cdot \gamma }_{xy}^{2}}$$
  The strain gradient on the surfaces is defined using MatLab R2024a’s gradient function [45], which is applied to the values of the $\varepsilon_{eq}$ distributions and to the coordinates of the corresponding element centroids. The two outputs of the gradient function are the gradient distributions along the local x-direction (${grad}_{X}$) and y-direction (${grad}_{Y}$), considering the coordinate system shown in Figure 1. To account for the effects in both directions, the magnitude is evaluated using Equation (12).
  (12) $${grad}_{tot}=\sqrt{{\left({grad}_{X}\right)}^{2}+{\left({grad}_{y}\right)}^{2}}$$
III. Clustering of constant gradient areas: For each surface where the distribution of ${grad}_{tot}$ was calculated, the areas with elements characterized by a constant ${grad}_{tot}$ value are identified using MatLab’s dbscan function [46].
IV. Evaluation of sensor removal: The final step in the gradient algorithm involves identifying surface locations where a sensor is not required. The principle behind this step is to evaluate the removal of one sensor at a time on each element of the sensor-applicable surfaces, following a specific sequence of analysis and separately for each surface. To represent the removal of a sensor from the surface, it is assumed that the corresponding value is absent from the $\varepsilon_{eq}$ distribution. Subsequently, the missing data are interpolated using polynomial functions. The accuracy of the resulting new distribution $\varepsilon_{eq,al}$ is assessed against the original $\varepsilon_{eq}$ values by calculating the maximum relative error $\left({err}_{rel,max}\right)$ and root mean square error $\left({RMSE}_{\varepsilon }\right)$, as defined in Equations (13) and (14). Both parameters must be lower than their respective acceptance thresholds, ${err}_{rel,limit}$ and ${RMSE}_{\varepsilon ,limit}$, in order for the removed sensor to be considered non-essential for interpolating the field $\varepsilon_{eq}$. The threshold values, ${err}_{rel,limit}$ and ${RMSE}_{\varepsilon ,limit}$, are estimated through an iterative process that involves repeated applications of the gradient algorithm, aimed at optimizing the filtering of sensor locations. Assigning threshold values that are too low could compromise the collection of an adequate number of relevant locations, while high values could limit the effectiveness of the filtering by including unnecessary locations. The optimal values of ${err}_{rel,limit}$ and ${RMSE}_{\varepsilon ,limit}$ are provided in Table 2.

(13) $${err}_{rel,max}={\left[{\left(\frac{\varepsilon_{eq,al}-\varepsilon_{eq}}{\varepsilon_{eq}}\right)}_{j}\right]}_{max}$$

(14) $${RMSE}_{\varepsilon }=\sqrt[{2}]{\frac{1}{N_{el}}\cdot \sum_{i=1}^{N_{el}}{\left(\varepsilon_{eq,al}-\varepsilon_{eq}\right)}_{j}^{2}}$$

where $N_{el}$ is the element count of the analysed surface, while j is the element index.

**Table 2:** Threshold values associated with Equations (13) and (14).

Maximum Threshold	Assigned Value
${err}_{rel,limit}$	0.1 [−]
${RMSE}_{\varepsilon ,limit}$	$4.43\cdot 10^{-6}\;[mm]$

This procedure is iterated for each element of the surface; if it is determined that the sensor associated with the specific element should not be part of the sensor network, it is excluded a priori from subsequent iterations. The analysis sequence for each surface is determined by its respective ${grad}_{tot}$ distribution. The process begins with the area that has the minimum ${grad}_{tot}$ value (defined in step III), which is conceptually considered most favorable for sensor removal, and then progresses in ascending order, progressively evaluating all elements in the examined surface areas. In cases where multiple areas have the same ${grad}_{tot}$ value, the analysis is conducted for each area in descending order based on the number of included elements. Within each area, the elements are analyzed according to their proximity to the centroid of that area, as the elements closest to the center in a constant gradient zone are expected to yield the most accurate interpolation of the missing data.

#### 4.3.2. Multi-Objective Function

The set of locations obtained from the gradient algorithm cannot be directly implemented as the sensor network for the inverse model. These results do not consider the practical limitations and constraints that should be considered in the installation of a sensor network layout. For the sensorization of a large structure such as a containership, it is preferable to adopt an FOP that is as regular as possible. This choice is advantageous both for practical constraints, such as the maximum number of acquisition systems that can be implemented, and to simplify the installation of the fiber optic cables, reducing positioning errors [47]. Based on these considerations, the data provided by the gradient algorithm are used as a reference for defining the FOP.

Regardless of the FOP proposed for the SHM system, it must be optimized to identify the most suitable configuration to meet the following objectives:

(1) Reduction in the cost associated with the SHM system.
(2) Minimization of the error associated with the full field of displacement reconstructed by the iFEM.

The quality of the results obtained from the iFEM approach is crucial for providing an accurate assessment of the ship’s health. At the same time, without limiting the costs for the implementation of SHM, the entire monitoring process would not be feasible.

This paragraph introduces the multi-objective function, expressed by Equation (15), which can weigh both aspects to identify the optimal configuration of the designed FOP.

(15) $$\Psi (n)=\frac{C(n)}{C_{min}}+\frac{RMSE(n)}{{RMSE}_{min}}$$

where the different terms of the equation are presented and described in Table 3.

The sensor network used for SHM is directly linked to the quality of the iFEM results. RMSE(n) serves as the reference parameter for assessing the overall error in the iFEM reconstruction of the displacement field.

The RMSE(n) is calculated using Equation (16), where N is the number of nodes in both the direct and inverse models (both models share the same mesh) and i represents the index of each specific node. $U_{mag,iFEM}$ and $U_{mag}$ are, respectively, the displacement magnitudes at the $i^{th}$-node from the iFEM reconstruction field and the direct model’s FEA results.

(16) $$RMSE(n)=\sqrt[{2}]{\frac{1}{N}\cdot \sum_{i=1}^{N}{\left(U_{mag,iFEM}-U_{mag}\right)}_{i}^{2}}$$

In addition to accuracy, a complementary aspect is the evaluation of costs associated with the SHM system, which include sensor acquisition, installation, and monitoring equipment [7]. Due to limited data availability, an approximate cost estimate is provided in Table 4. These values were determined based on the type of sensors used, specifically DTG, and the estimated time required for designing and implementing the sensor network. The overall estimate was formulated assuming a one-year timeframe for installing the sensors on the containership, considering operational complexities and the specific requirements associated with this application.

The total cost C(n) of the sensor network is derived from the weighted sum of the individual components listed in Table 4, as described in Equation (17):

(17) $$C(n)={n\cdot (C}_{s}+C_{str})+C_{pd}+C_{pi}$$

**Figure 8:**
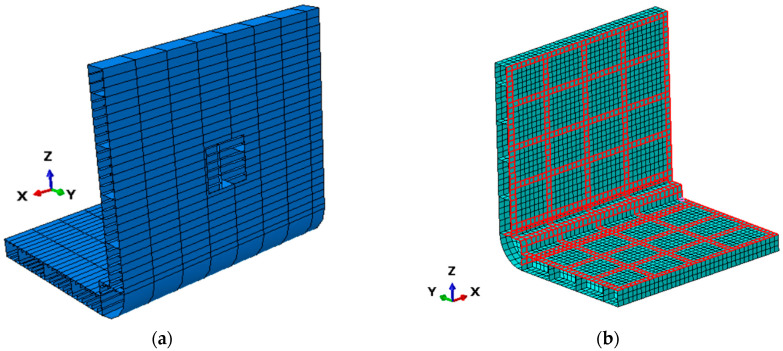
Data from the damage localization study: (**a**) structural model with a representative damage, (**b**) sensor network.

The values of RMSE(n) and C(n) enable a quantitative assessment of the targeted objectives (1) and (2). To derive a balanced solution that accounts for both aspects simultaneously, the multi-objective function Ψ(n) incorporates the normalization factors ${RMSE}_{min}$ and $C_{min}$ to allow a direct comparison between RMSE(n) and C(n). ${RMSE}_{min}$ and $C_{min}$ are the resulting values, calculated using Equations (16) and (17), representing the iFEM results derived from two different sensor networks, each optimized, respectively, for objectives (1) and (2). The normalization factors are specific to the present case and cannot be generalized a priori for all situations. The quantity $C_{min}$ is defined as the cost associated with the least expensive configuration of the proposed FOP, specifically the one requiring the fewest sensors. Regarding the ${RMSE}_{min}$ factor, it is defined as the RMSE(n) of a sensor network that can ensure, in this case, a 95% probability of detection (POD). To determine the value of ${RMSE}_{min}$, the results of an analysis conducted for damage detection in SHM on the same ship model were considered. The model used for that purpose, illustrated in Figure 8a, corresponds to a simplified inverse model, considering only half of the structure. Out of 100 analyzed cases, each characterized by a different simplified damage location, a Gaussian-distributed error (with a mean of 5% and a standard deviation of one) was introduced to the measurements collected by the fiber optic sensor network presented in Figure 8b. This was performed to realistically simulate the performance of strain acquisition systems. The results indicated damage detection in 95% of the cases, thus defining the POD value associated with the sensor network in Figure 8b. Both normalization factors are reported in Table 5.

#### 4.3.3. Sensor Network Optimization

**Figure 9:**
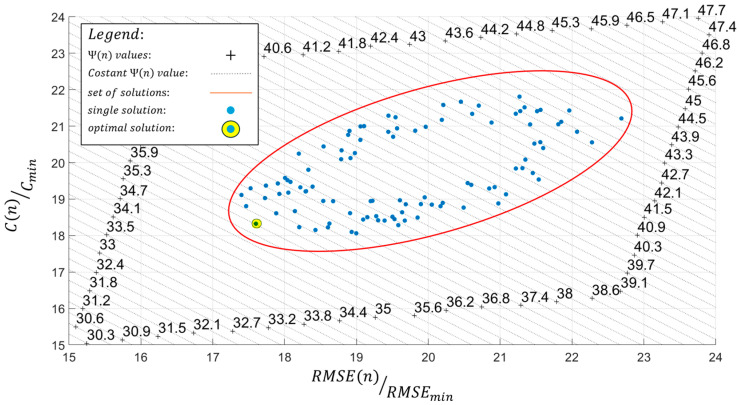
Graphic optimization example.

In this paper, the optimal configuration of the FOP for the sensor network is the one that minimizes the multi-objective function Ψ(n). This solution is determined graphically by plotting the results of all possible configurations of the selected FOPs on a graph of $\left(RMSE(n)/{RMSE}_{min},C(n)/C_{min}\right)$ values and identifying the solution that is furthest along the direction of minimizing Ψ(n) values, as illustrated in the example of Figure 9.

### 4.4. Internal Force Reconstruction

**Figure 10:**
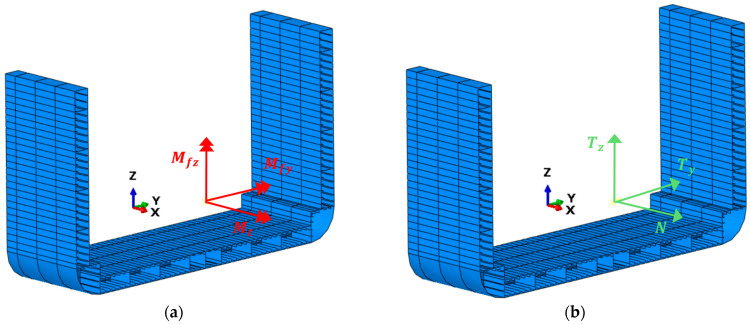
Example of internal forces to which a cross-section can generally be subjected: (**a**) moments, (**b**) forces.

Within the proposed framework, in addition to reconstructing the full field of displacement and strain, the procedure for calculating internal forces at several cross-sections of the containership is performed. Following the optimization of the sensor network and its implementation on the inverse model, the procedure described in Section 2 can be applied, using the reconstructed field $\varepsilon_{iFEM}$ through the iFEM approach, to each cross-section of the analyzed structure. Regardless of loading conditions, it allows for the estimation of all possible internal forces that a cross-section may generally experience (Figure 10a,b). Among the infinite cross-sections of the containership, for simplicity, this paper analyses only those listed in Table 6, with their coordinates referenced to the coordinate system in Figure 10. For a given cross-section of interest in the inverse model, the calculation of the associated internal forces, illustrated in the example in Figure 10, involves summing the contributions of all elements intersected by the cross-section.

From the results of the iFEM approach, the field $\varepsilon_{iFEM}$ is used as input in Equation (9) to calculate all the generalized stresses associated with each element of the analyzed cross-sections. Finally, the internal forces are obtained through Equation (18), as the sum of the individual contributions from each element of the cross-section, where ${\left[N_{x},N_{y},N_{xy},M_{x},M_{y},M_{xy},Q_{x},Q_{y}\right]}_{i}^{T}$ and $l_{i}$ correspond to the vector of generalized stresses and the length of the $i^{th}$ element. The only exception concerns the moment $M_{fx}$, for which the traditional plate/shell theories, on which Equation (9) is based, do not provide the corresponding generalized stress. As an alternative, this moment is calculated as the derivative along the x-axis of the $T_{z}$ distribution, as a function of the $\bar{x}$-coordinate of the cross-section under consideration, with respect to the coordinate system in Figure 10.

(18) $$N=\sum_{i=1}^{N_{el}}{l_{i}\cdot \left(N_{x}\right)}_{i}\;T_{y}=\sum_{i=1}^{N_{el}}{l_{i}\cdot \left(N_{xy}\right)}_{i}\;T_{z}=\sum_{i=1}^{N_{el}}{l_{i}\cdot \left(Q_{x}\right)}_{i}\;M_{fz}=\frac{\partial T_{z}\left(\bar{x}\right)}{\partial x}\;M_{fy}=\sum_{i=1}^{N_{el}}{l_{i}\cdot \left(M_{x}\right)}_{i}\;M_{t}=\sum_{i=1}^{N_{el}}{l_{i}\cdot \left(M_{xy}\right)}_{i}$$

where $N_{el}$ is the number of elements at the cross-section analyzed.

## 5. Results and Discussion

**Figure 11:**
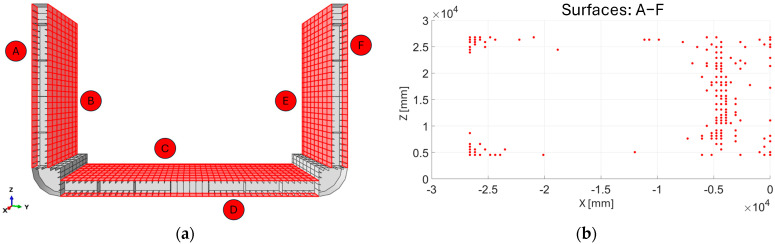

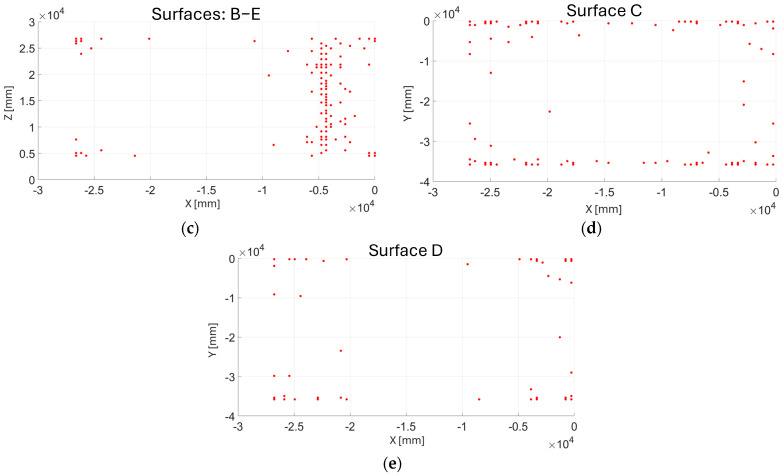
(**a**) Sensor-applicable surface indication. Gradient algorithm results for each sensor-applicable surface: (**b**) surfaces A–F, (**c**) surfaces B–E, (**d**) surface C, (**e**) surface D.

In this section, the results of the application of the framework are presented and discussed, following the same progression logic of the scheme reported in Figure 3. The results of the direct FEM model (Figure 6) define the strain field $\varepsilon_{eq}$, which is used as input in the gradient algorithm to identify, on each of the sensor-applicable surfaces highlighted in Figure 6d, the locations of the minimum number of elements in the inverse model that require a sensor in order to ensure an accurate extrapolation and reconstruction of the entire field $\varepsilon_{eq}$. The gradient algorithm provides the results presented in Figure 11b–e.

**Figure 12:**
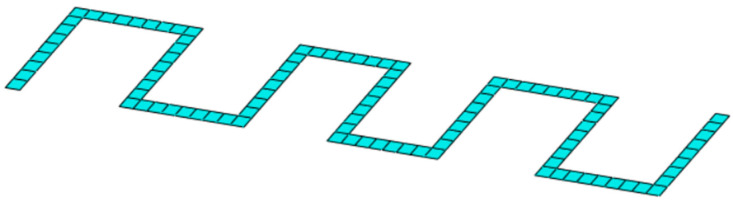
Example of a serpentine sensor pattern (at the centroid of each mesh element, there is an optical fiber measurement point).

As shown in Figure 11b–e, the locations (in red) identified by the algorithm cannot be directly implemented to create a real and practical sensor network, as sparse point distributions are observed. This limitation arises from the omission of practical aspects related to the operation of the optical fiber in the gradient algorithm. By using the results presented in Figure 11b–e as a reference, an FOP has been designed to consider the use and implementation of optical fibers. Specifically, a serpentine sensor pattern was chosen, as shown in Figure 12.

**Figure 13:**
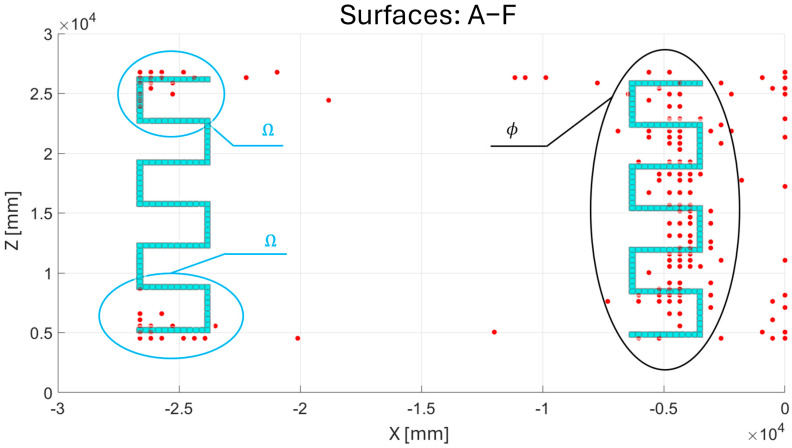
Set of positions ϕ, between coordinates X −7500 mm and −2500 mm, and set of positions Ω, between coordinates X −27,000 mm and −22,500 mm.

The choice of this specific pattern is intended to cover the set of positions ϕ shown in Figure 13. One important consideration is that the results generated by the gradient algorithm are based on the analysis of the strain field $\varepsilon_{eq}$, which represents all measurable strain components. Since optical fibers can detect only a single strain component, the adoption of a serpentine pattern was chosen to address this limitation. This configuration enables strain measurements in multiple directions, thereby ensuring a more comprehensive and accurate detection of the strain field. Moreover, the use of more regular patterns is better suited for monitoring large surfaces [47,48], such as those considered in Figure 6d. However, it is important to note that there are additional positions outside of the set ϕ. To account for these residual positions, indicated as sets Ω in Figure 13, the serpentine pattern has also been implemented for these regions. These observations are applicable not only to surfaces A and F in Figure 13 but also to the surfaces in Figure 11c, due to the similarity of the gradient algorithm’s results. 

**Figure 14:**
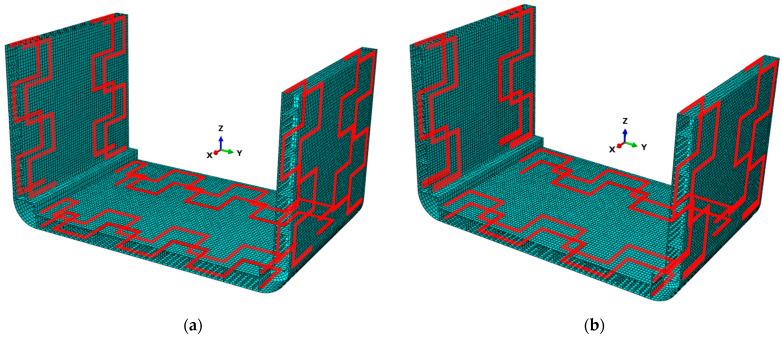
(**a**) Proposed FOP as a sensor network of the containership and (**b**) optimal configuration.

The proposed FOP, shown in Figure 14a, includes uniform extension of the serpentine pattern to the surfaces indicated in Figure 11d,e. This choice was made to ensure a regular arrangement of sensors across the entire container structure, facilitating installation. This extension is justified by the results shown in Figure 11d,e, which exhibit a random sensor arrangement with no defined trend, due to the uniform distribution of the measured deformations. As a result, there are no restrictions in the pattern selection for these surfaces.

**Figure 15:**
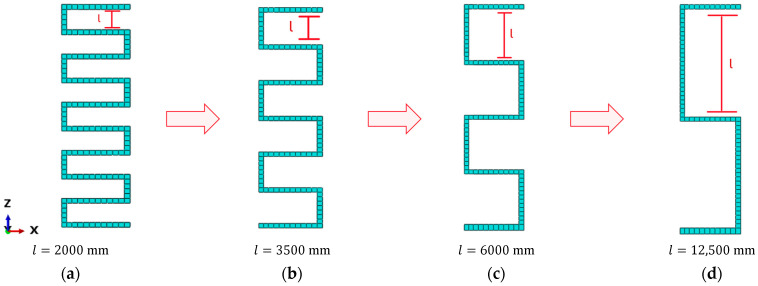
Serpentine patterns parameterized based on the distance l.

The proposed FOP as a sensor network needs to be properly parameterized to determine the optimal configuration. The parameter selected for the serpentine pattern’s parameterization is the distance l between the curves, as shown in Figure 15. For each configuration, the value of l between the curves is assumed to remain constant.

For the SHM of the containership, the optimal configuration of the pattern must ensure an accurate assessment of the structure’s health while maintaining economic sustainability.

In this case, the domain of possible configurations for the proposed FOP (Figure 15) is limited by the finite size of the mesh discretization in the inverse model. Each configuration is individually evaluated in terms of sensor cost and accuracy of $U_{mag,iFEM}$, assuming it as the sensor network on the inverse model within the iFEM approach. The cost and accuracy of the results are quantified using the parameters C(n) and RMSE(n), calculated according to Equations (16) and (17). Each configuration, along with the parameters C(n) and RMSE(n), and the number of sensors used n, is presented in Table 7. Among the available configurations, the optimal option for SHM is the one that minimizes the multi-objective function Ψ(n). This configuration is identified graphically by plotting the values reported in Table 7 on the RMSERMSEmin,CCmin plane, where each of the 25 points represents a possible configuration of the proposed FOP (Figure 14a).

**Figure 16:**
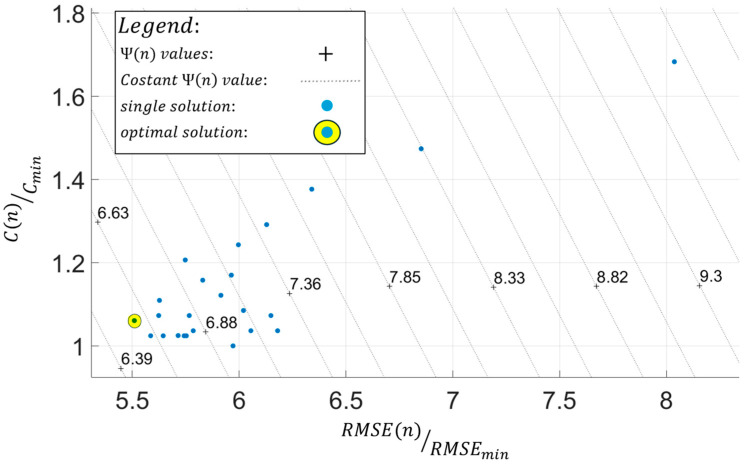
Results of the optimization procedure.

Once all terms of the multi-objective function Ψ(n) have been defined, the optimal configuration of the proposed FOP can be identified graphically in Figure 16 as the solution that minimizes this function.

As a result of the optimization procedure, the optimal configuration of the proposed FOP, identified with index 16 in Table 7, is characterized by an l value of 8000 mm and a Ψ(n) value of 6.57 and is illustrated in Figure 14b.

Referring to Figure 16 and Table 7, it can be observed that there is generally no clear proportionality between the parameters C(n) and RMSE(n). The difference between various configurations lies in the variable n; conceptually, increasing n should result in both an increase in C(n) and a reduction in RMSE(n). However, this relationship was not confirmed by the results shown in Table 7. The reason for this undefined trend lies in the RMSE(n) parameter, as C(n) has a direct proportionality with n according to Equation (17). Conversely, RMSE(n) does not display a linear proportionality, which may be attributed to the choice of RMSE(n) as the metric for evaluating iFEM results, as it is a global representative parameter that does not account for the actual distribution of error in the displacement field modulus.

**Figure 17:**
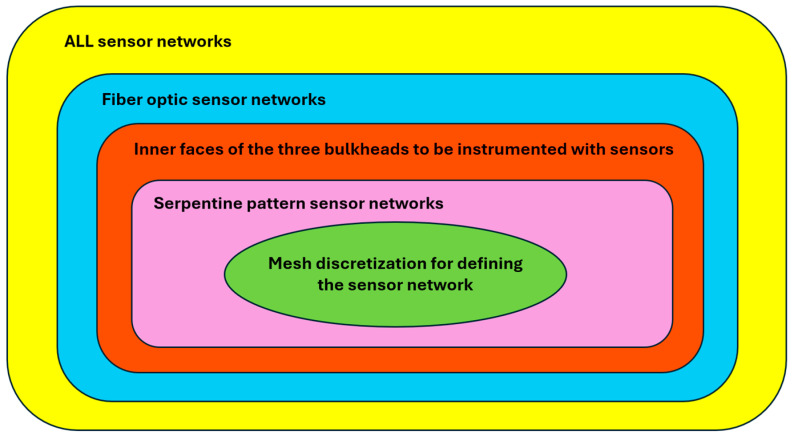
Filtering scheme for sensor network characteristics.

Another reason for this undefined trend is the limited set of configurations analyzed. In general, there are countless options for the sensor network to be implemented on the containership for SHM, but only a subset has been evaluated. As illustrated in Figure 17, the set of all possible sensor networks is progressively narrowed based on the specific characteristics of the case under consideration:

- The selection of a specific sensor type.
- The selection of areas to be instrumented with sensors.
- The choice of a specific sensor pattern.
- The mesh size of the inverse model.

As a result, although the number of available sensor networks is reduced, this provides a more targeted approach to the problem, ruling out inefficient or infeasible solutions from the outset and reducing analysis time.

**Figure 18:**
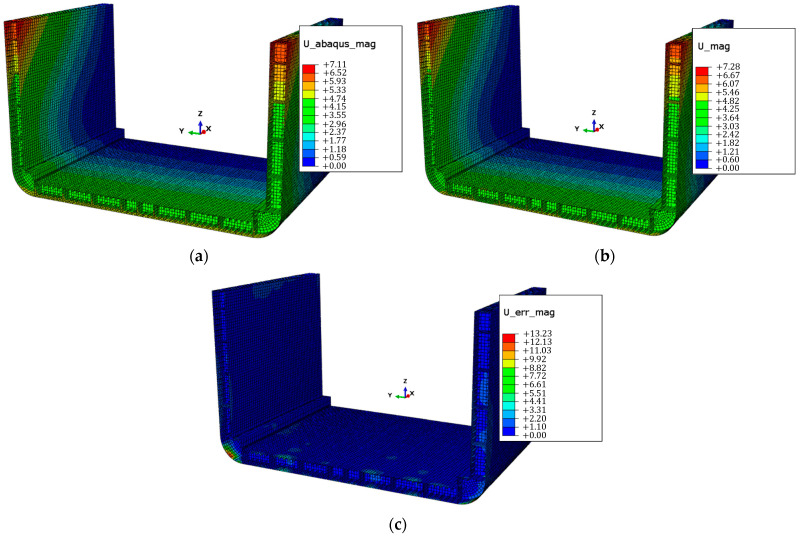
Comparison of displacement fields: (**a**) U_abaqus_mag: magnitude displacement field of direct model’s FEA results (in mm); (**b**) U_mag: magnitude displacement field reconstructed by iFEM (in mm); (**c**) U_err_mag: percentage difference between fields (**a**,**b**).

**Figure 19:**
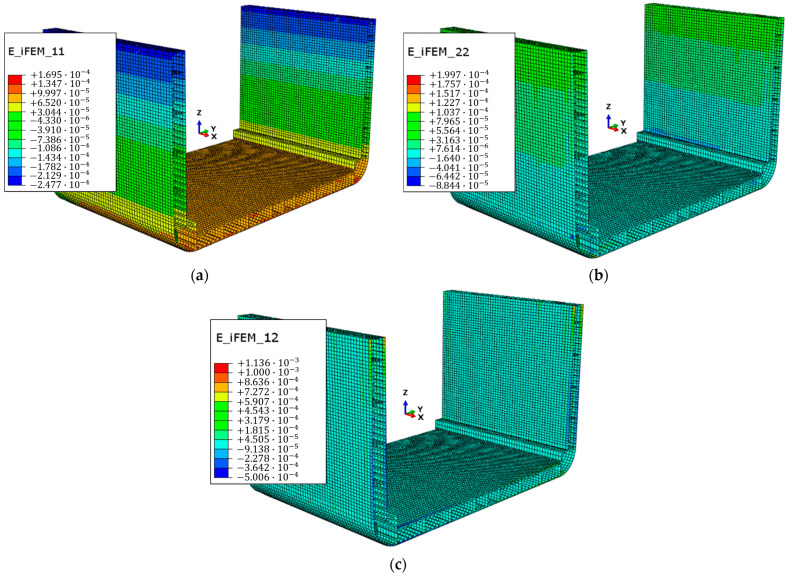
iFEM reconstructed strain fields: (**a**) E_iFEM_11, normal strain field along x-direction; (**b**) E_iFEM_22, normal strain field along y-direction; (**c**) E_iFEM_12, shear strain field on x, y-plane.

After identifying the optimal configuration of the proposed FOP, it is implemented as the sensor network for the inverse model. The iFEM approach is then applied to obtain the displacement and strain fields, shown, respectively, in Figure 18 and Figure 19.

**Figure 20:**
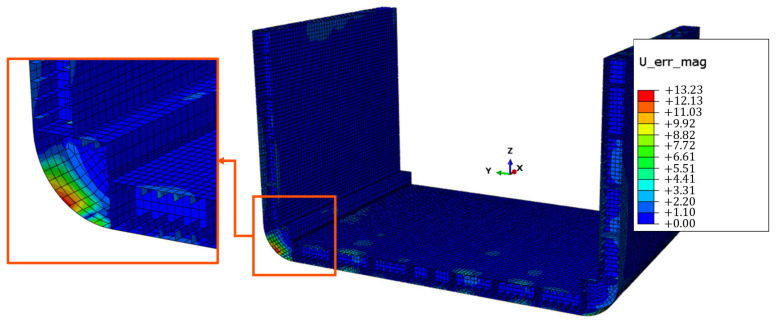
Peak zones of the field U_err_mag (percentage difference between fields in Figure 18a,b).

The results in Figure 18 demonstrate how the sensor network shown in Figure 14b enables an accurate reconstruction of the displacement field, with the error distribution in Figure 18c estimated as the percentage difference between the magnitude displacement field from the iFEM reconstruction and the FEA results of the direct model. It can be observed that the error distribution in the magnitude displacement field shows very low values, below 2%, except for the peak zones highlighted in Figure 20, where the error rises to 13.23%. It is important to note that Figure 20 shows a close-up of a portion of the model, but this observation also applies to the symmetrically opposite surfaces relative to the x-z plane.

This distribution can be attributed to the extrapolation method adopted, as the peak error zones of Figure 20 were excluded from the set of surfaces on which extrapolation of missing data is performed. This indicates that attention should be focused on the extrapolation technique to be used for these specific surfaces, as the polynomial-based approach applied to the rest of the structure proved ineffective here. Despite the large scale of the structure, the designed sensor network allows for a highly accurate reconstruction of both displacement and strain fields, with a minimal number of strain measurements. This result highlights the practicality and precision of the network, making it a viable solution for SHM application on the containership.

Subsequently, the results in Figure 19 were used in the procedure described in Section 2 and Section 4.4 for calculating the internal forces at the cross-sections listed in Table 6. The error is evaluated as the percentage difference between the internal force $M_{fy}$ reconstructed by the applied procedure and that obtained from the direct model. The remaining internal forces are not included, as they are close to zero in both reconstructions. The results, presented in Table 8, demonstrate the accuracy of the internal force calculations for the different cross-sections analysed, with a maximum error below 3.5%.

Despite the relatively low number of sensors applied in the benchmark case, the quality of the iFEM application results shows that the procedure for calculating internal forces remains equally accurate. All the results described confirm the effectiveness of the proposed framework in the field of SHM. The framework enables the analysis of real and complex cases, such as the containership under study, with comprehensive management of the entire sensorization process: from creating a sensor network to obtaining accurate results that are valuable for monitoring.

## 6. Conclusions

This paper proposes a comprehensive framework for implementing hull structural health monitoring on the midsection of a containership using the iFEM approach. This study addresses a significant gap in the literature, where an optimized, sensor-based monitoring strategy for complex naval structures, such as containerships, is largely absent. Unlike typical applications of iFEM, which may be limited in scope or complexity, the proposed method is designed to precisely reconstruct the displacement and strain fields, as well as internal cross-sectional forces, in complex structures like containerships. A key aspect of the framework is the design of a high-performance sensor network with a minimal number of sensors, strategically placed to capture critical data with high accuracy. The initial sensor layout is informed by a gradient trend analysis and further refined through a multi-objective optimization process that balances cost efficiency with monitoring accuracy.

Specifically, the framework started by developing a finite element analysis of the containership structural model, obtaining a resulting strain field that was then processed using a gradient analysis algorithm to identify the reference positions needed for the creation of an optical fiber sensor pattern to be implemented as a sensor network. The optical fiber sensor pattern was then parametrized and optimized through a multi-objective function that balances the costs associated with the structure’s monitoring system and the quality of the results obtained from the iFEM approach.

Once the sensor network was optimized, the iFEM approach was applied; from the reconstructed deformation field, the internal forces reconstruction formulation was then implemented on the cross-sections of interest of the containership.

Within the framework, particular attention was given to the feasibility of the proposed solutions. Specifically, in the design of the sensor network, environmental challenges related to sensor placement were considered, leading to the decision to place the strain sensors exclusively on the inner side of the ship’s bulkheads.

Following the implementation of the framework, it was found that, despite the relatively small number of sensors distributed on a structure as large as the containership’s, a highly accurate reconstruction of the deformation fields, displacements, and internal forces along the structure was achieved. This result highlights the effectiveness of the sensor network design procedure for the SHM of the containership.

Within the present framework, only the longitudinal vertical bending action has been applied to the model for simplicity. Future studies will expand the framework to account for all global and local loads to which the ship may be subjected.

The proposed framework demonstrates significant potential and adaptability for future real-world applications, offering a fast and efficient method for creating high-performance sensor networks, real-time monitoring through the iFEM approach, and internal force reconstruction formulation. Ultimately, it contributes to improving safety and the effectiveness of managing maritime structures.

## Figures and Tables

**Table 1:** Matrix W weights from case to case.

Case	Weight Coefficient
Measured strain component	1
Extrapolated strain component	$10^{-1}$
No extrapolated and no measured strain component	$10^{-4}$

**Table 3:** Description of the different terms of Equation (15).

Term	Description	Unit
n	Number of sensors employed	−
C(n)	Sensor network’s overall cost	€
$C_{min}$	Minimum investment cost for a sufficiently performing sensor network	€
RMSE(n)	Root mean square error of the displacement magnitude field result, related to a sensor network	mm
${RMSE}_{min}$	Minimum root mean square error of the displacement Magnitude field for a reference sensor network	mm

**Table 4:** Sensor network-related costs.

Term	Cost Type	Value
$C_{s}$	Sensor purchase cost	$30\;\left[€/n\right]$
$C_{str}$	Sensor instrumentation cost	$75\;\left[€/n\right]$
$C_{pd}$	Engineering personnel cost	$150000\;\left[€\right]$
$C_{pi}$	Manual labor cost	$100000\;\left[€\right]$

**Table 5:** Normalization factors assigned to the multi-objective function Ψ(n).

Normalization Factor	Assigned Value
Measured strain component	380620 €
${RMSE}_{min}$	0.942 mm

**Table 6:** Coordinates of the analyzed cross-sections.

Cross-section x-coordinate $\left[mm\right]$	−25,000	−20,000	−15,000	−10,000	−5000

**Table 7:** Parameters of the different configurations of the FOP in Figure 14.

Index	$l\;\left[mm\right]$	$n\;\left[-\right]$	$C(n)\;\left[€\right]$	$RMSE(n)\;\left[mm\right]$	Index	$l\;\left[mm\right]$	$n\;\left[-\right]$	$C(n)\;\left[€\right]$	$RMSE(n)\;\left[mm\right]$
1	500	5028	777,940	0.095	14	7000	1508	408,340	0.058
2	1000	3718	640,390	0.076	15	7500	1508	408,340	0.053
3	1500	2960	560,800	0.065	16	8000	1464	403,720	0.052
4	2000	2608	523,840	0.060	17	8500	1376	394,480	0.057
5	2500	2300	491,500	0.058	18	9000	1376	394,480	0.058
6	3000	2124	473,020	0.056	19	9500	1376	394,480	0.054
7	3500	1992	459,160	0.054	20	10,000	1334	390,070	0.054
8	4000	1860	445,300	0.056	21	10,500	1332	389,860	0.054
9	4500	1816	440,680	0.055	22	11,000	1332	389,860	0.053
10	5000	1684	426,820	0.056	23	11,500	1332	389,860	0.053
11	5500	1640	422,200	0.053	24	12,000	1332	389,860	0.054
12	6000	1552	412,960	0.057	25	12,500	1244	380,620	0.056
13	6500	1508	408,340	0.054					

**Table 8:** Results of $M_{fy}$ reconstruction from iFEM results.

Cross-Section $x-\mathrm{Coordinate}\;\left[mm\right]$	$M_{fy}$ from Direct Model’s $\mathrm{FEA}\;\mathrm{Results}\;\left[Nmm\right]$	$M_{fy}$ $\mathrm{Reconstruction}\;\mathrm{from}\;\mathrm{iFEM}\;\mathrm{Results}\;\left[Nmm\right]$	Percentage Difference %
−25,000	$8.999\cdot 10^{11}$	$9.118\cdot 10^{11}$	1.32
−20,000	$9\cdot 10^{11}$	$9.215\cdot 10^{11}$	2.39
−15,000	$9\cdot 10^{11}$	$9.293\cdot 10^{11}$	3.25
−10,000	$9\cdot 10^{11}$	$9.294\cdot 10^{11}$	3.27
−5000	$9.004\cdot 10^{11}$	$9.282\cdot 10^{11}$	3.09

## Data Availability

The data given in this article are the data supporting the results of this study and are available upon request.
